# Influential Cognitive Processes on Framing Biases in Aging

**DOI:** 10.3389/fpsyg.2018.00661

**Published:** 2018-05-11

**Authors:** Alison M. Perez, Jeffrey Scott Spence, L. D. Kiel, Erin E. Venza, Sandra B. Chapman

**Affiliations:** ^1^Advanced Technology Laboratories, Lockheed Martin, Arlington, VA, United States; ^2^School of Behavioral and Brain Sciences, The Center for BrainHealth, The University of Texas at Dallas, Dallas, TX, United States; ^3^School of Economic, Political and Policy Sciences, The University of Texas at Dallas, Richardson, TX, United States

**Keywords:** framing effects, aging, cognition, decision-making, strategic attention

## Abstract

Factors that contribute to overcoming decision-making biases in later life pose an important investigational question given the increasing older adult population. Limited empirical evidence exists and the literature remains equivocal of whether increasing age is associated with elevated susceptibility to decision-making biases such as framing effects. Research into the individual differences contributing to decision-making ability may offer better understanding of the influence of age in decision-making ability. Changes in cognition underlying decision-making have been shown with increased age and may contribute to individual variability in decision-making abilities. This study had three aims; (1) to understand the influence of age on susceptibility to decision-making biases as measured by framing effects across a large, continuous age range; (2) to examine influence of cognitive abilities that change with age; and (3) to understand the influence of individual factors such as gender and education on susceptibility to framing effects. 200 individuals (28–79 years of age) were tested on a large battery of cognitive measures in the domains of executive function, memory and complex attention. Findings from this study demonstrated that cognitive abilities such as strategic control and delayed memory better predicted susceptibility to framing biases than age. The current findings demonstrate that age may not be as influential a factor in decision-making as cognitive ability and cognitive reserve. These findings motivate future studies to better characterize cognitive ability to determine decision-making susceptibilities in aging populations.

## Introduction

In our society, older adults continue to face many complex decisions regarding financial investments and retirement choices. Decisions made by older adults will predictably influence the economic resources of our world, given they represent a large proportion of not only our nation but the world’s population (Vaupel, 2010). Research across the adult lifespan has suggested age-related changes are shown in decision-making abilities (Denburg et al., 2005; Weller et al., 2011). Moreover, the American Association of Retired Persons reports on the increased vulnerability of older adults to financial scams, making them a target of financial abuse (Kirchheimer, 2011). Taken together, understanding changes that occur with age in the ability to make “reasoned” decisions related to allocation of financial resources is of public health and national policy concern.

One of the most important elements underlying commonly occurring decisions is the element of risk, particularly in monetary-based decisions. To investigate risk in decision-making, this study utilized a risky-choice financial framing task. Risky-choice framing paradigms present individuals with decisions in terms of sure options versus gamble options involving risk. The method by which the sure options are framed can create framing effects, namely, the propensity of individuals to engage in risky behavior (Takemura, 1993). Framing effects are a common heuristic bias which arise when the context of decision options influence or change the choices people make, whether being presented as a gain or a loss (Tversky and Kahneman, 1981; Kahneman and Tversky, 1990).

Research examining the influence of aging on framing effects has found mixed results with findings ranging from older adults performing better, worse, or similarly when compared to young adults. Given the inconsistencies of age-related differences in decision-making behavior, researchers have increasingly theorized that cognitive factors may predict framing effects in older adults, perhaps better than age alone. The present study examined three primary issues. First, the influence of age on decision-making performance on a monetary-based framing paradigm across a broad age range (28–79 years) of cognitively normal adults. Second, this study assessed how age influences cognitive processes that may be involved in decision-making. Third, we investigated the effects of individual factors such as cognition, education, and gender on monetary-based choices.

Research has shown that the influence of age on susceptibility to framing biases is inconsistent (Loke and Tan, 1992; Kim et al., 2005; Mikels and Reed, 2009; Strough et al., 2011). Indeed, studies that show age to be a factor in predicting framing effects have qualified the results, suggesting age effects on decision-making are mediated by cognitive ability (Mata et al., 2007, 2011; Mata and Nunes, 2010; Thomas and Millar, 2011; Del Missier et al., 2012). To date, only a handful of researchers have empirically assessed the possible connections between cognitive abilities and age differences in decision-making as measured framing effects (Finucane et al., 2005; Del Missier et al., 2010; De Bruin et al., 2012; Li et al., 2013; Pu et al., 2017).

Research that has looked at cognitive ability in relation to framing effects have found higher cognitive performance on measures of reasoning, inhibition, verbal intelligence and memory are associated with reduced framing effects in older adults (De Bruin et al., 2007, 2012; Cokely and Kelley, 2009; Del Missier et al., 2012). Another study found that complex attention significantly predicted framing effects in younger adults (18–22 years old) (Kuo et al., 2009). It is unclear whether complex attention remains a factor in predicting framing effects with increasing age. With regard to memory, Whitney et al. (2008) demonstrated that increased memory capacity predicted increased ability to overcome framing biases in adults (18–24 years old). Studies that examined cognition in relation to framing effects in both younger and older adults found better cognitive abilities in inhibition, reasoning and verbal intelligence to relate to overcoming framing biases (Del Missier et al., 2010; De Bruin et al., 2012; Li et al., 2013). Such evidence is beginning to suggest that some cognitive abilities can influence susceptibility toward framing biases across adulthood.

One important factor to take into account in decision-making is the cognitive decline that has been reported with increased age (Andrews-Hanna et al., 2007; Salthouse, 2009). Indeed, a number of cognitive aging studies have documented normal age-related deleterious effects on cognition, starting to emerge around 40 years of age (Salthouse and Babcock, 1991; Salthouse, 1992, 1996, 2009; Levy, 1994; Craik, 2000; Park et al., 2002; Li et al., 2004; Worthy et al., 2011). The key domain of this age-related decline tends to affect performance on measures of executive function, defined as cognitive processes that direct and control behavior (Miller and Cohen, 2001). Declines in the cognitive ability of older adults raise the concern that older adults may not maintain capacity to make optimal decisions in everyday life.

One limitation in the existing evidence on the influence of age on framing effects is that few studies have examined framing effects across contiguous age cohorts (Strough et al., 2011). Instead, the majority of studies have investigated the influence of framing biases on decision-making by comparing groups of older adults to groups of younger adults (i.e., college aged students) with a gap excluding middle-aged adults (Mayhorn et al., 2002; McArdle et al., 2002; Kovalchik et al., 2005; Rönnlund et al., 2005). Declines in decision-making reportedly become apparent starting in the fifth decade of life (or middle-age) and worsen in older adulthood, as reflected in real-life measures such as suboptimal credit use (Agarwal et al., 2009). At this mid to late life stage, insidious losses in cognitive abilities, such as executive functions of fluency and working memory in addition to processing speed, emerge and continue to decline with advancing age (Salthouse, 2009). Another limitation of current evidence is the limited range of measures adapted to examine the effects of cognitive capacity in relation to framing effects generally. Specifically, research has addressed the influence of cognitive abilities on framing effects in a narrow focus of cognitive domains.

In sum, this work expands on prior evidence in three major ways providing the following hypotheses. First, this study investigated framing effects across a continuous five-decade adult age span. We hypothesized that age would be negatively associated with framing effects based on prior evidence suggesting older individuals would tend to be more risk-averse when facing both gains and losses than younger adults, thus older adults will show fewer framing effects (Mikels and Reed, 2009; Best and Charness, 2015). Second, this study measured age effects on our cognitive measures to confirm prior evidence implicating generalized age-related declines on measures of executive function, memory and complex attention in order to verify whether the previously established age-related pattern of cognitive loss was also identified in a group that was well-screened as being cognitively normal. We hypothesized that age-related cognitive declines would be manifested across the cognitive measures based on extant evidence suggesting normal age losses on measures of executive function, memory and complex attention as well as slower reaction times in most cognitive tests (Park et al., 2002; Salthouse, 2009; Schaie and Willis, 2010). Third, this study uniquely examined a wide array of cognitive abilities of executive function, memory and complex attention as well as individual factors such as education and gender to understand their relationship to framing effects in a large adult population. We hypothesized that gender may influence framing effects, especially in a financial framing paradigm, based on previous evidence that women are more risk-averse in financial decision-making than men (Levin et al., 1988; Johnson and Powell, 1994; Powell and Ansic, 1997; Austad, 2006).

## Materials and Methods

### Participants

Two hundred adults, between the ages of 28–79, were recruited from the Dallas–Fort Worth area to participate in a decision-making study. All participants provided written informed consent in accordance with guidelines provided by The University of Texas at Dallas Institutional Review Board and the Declaration of Helsinki. Participants were all native speakers of English with a minimum of high school education. Exclusion was not based on gender or race although 95.91% of our sample was Caucasian. Our study screened and excluded participants with cognitive and medical problems, such as complaints of poor memory, stroke, major psychiatric illness, uncorrected hearing/vision problems, and chronic medical conditions. Screening was rigorous given the study goals of investigating decision-making behavior in a cognitively normal adult population. Screening measures were employed to determine basic cognitive skills using the Montreal Cognitive Assessment (MoCA) to estimate general cognitive abilities as well as the Beck Depression Inventory-II (BDI-II) to exclude for depression and a medical history form to screen for chronic conditions and difficulties completing daily activities (Beck et al., 1996; Nasreddine et al., 2005). Only those who received a score of 25 or higher out of 30 on the MoCA and a score of 13 or lower on the BDI (suggesting fewer endorsed depression symptoms) were included in the study. Among the age groups, 47 participants were between the ages of 28–44 representing our younger adults group, 112 participants were between the ages of 45–65 representing our middle-aged adult group, and 46 participants were between the ages of 66–79 representing our older adult group. Demographic characteristics of the sample of participants are shown in **Table 1**.

**Table 1:** Demographic and test measures.

Measure	Mean (SD)	Range
Age	55.97 (+/-11.92)	28–79
Gender	108 Male	
Education	17.01 (+/-2.26)	12–31
BDI-II	3.83 (+/-3.72)	0–13
Cognitive reflection test	1.19 (+/-1.09)	0–3

### Measures

Participants completed approximately three hours of cognitive testing scheduled at a convenient time for their schedule. Testing was conducted by researchers at the Center for BrainHealth, The University of Texas at Dallas in a private testing room and breaks were given during the testing session as needed by the participant. After the in-person screening which took approximately 10 min, participants began with cognitive testing if their screening scores passed the threshold mentioned previously. Cognitive testing included the following measures of executive function, memory, and complex attention. Descriptions of these measures are delineated in **Table 2**.

**Table 2:** Measurements of cognition.

Cognitive domain	Cognitive ability	Measures	Time to complete	Description
Executive function	Abstraction	Similarities subtest (WAIS-III) (Wechsler, 1997)	10 min	Participants are asked to create meaningful similarities between pairs of words
	Verbal fluency	COWA (Schum et al., 1989)	5 min	Name as many words that begin with a given letter within one minute.
	Inhibition	Delis–Kaplan executive function system color word interference test	5 min	Read color blocks, read words denoting colors, read words printed in colors not associated with the printed word to measure both processing speed and inhibition
	DKEFS sorting	Delis–Kaplan executive function system sorting test (Shunk et al., 2006)	20 min	Sort two sets of cards as many ways as possible, recognize the sort the tester provides with the same card sets. Measures verbal and nonverbal executive function
	Strategic control	Strategic Learning Task (Hanten et al., 2004)	15 min	Participants are asked to recall words from a list presented to them. Words are given high and low point values. Participants points by remembering words.
Memory	Immediate and delayed memory	Logical memory subtest I and II (WMS-III) (Wechsler, 1997)	10 min	Participants are asked to recall details of two short stories once immediately after hearing the stories and again after a 25 minute delay
	Working memory	Digit backward (WASI-III) (Wechsler, 1999)	10 min	Orally recall number strings read aloud in backward order
Complex attention	Processing speed	Trails A (Reitan, 1958)	5 min	Connect a set of numbers in ascending sequence as quickly as possible
	Switching	Trails B (Reitan, 1958)	5 min	Connect a set of altering numbers and letters in ascending sequence as quickly as possible.
	Attention	Digit forward (WASI-III) (Wechsler, 1999)	10 min	Orally recall a string of numbers read aloud in the same order

### Framing Task

**FIGURE 1:**
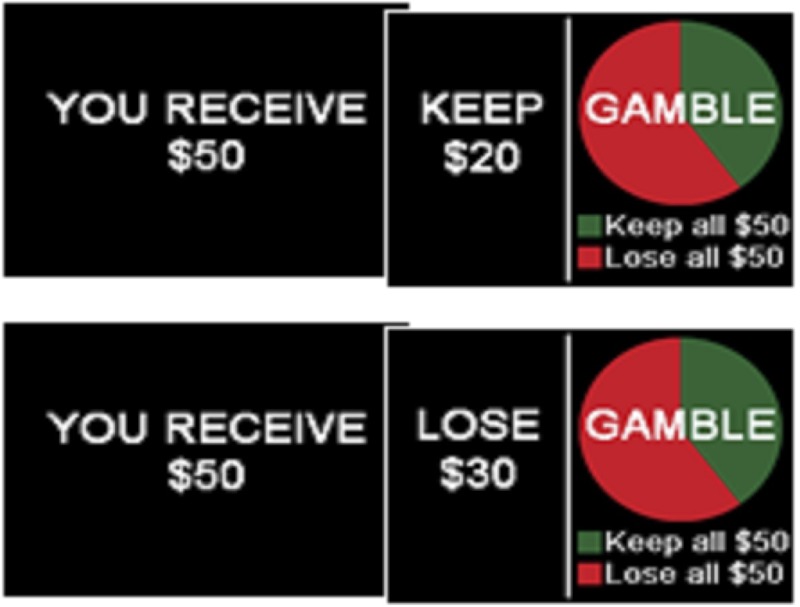
Financial risky-choice framing paradigm. The decision-making task used in this study was adapted from De Martino et al. (2006) by using American dollar amounts instead of representing monetary values in euros. Participants were required to choose between keeping/losing a portion of the initial endowment of each trial or choosing to gamble on the initial endowment to either win or lose the entire amount, with the probability of winning represented by the pie chart on the right. The two options are balanced in that the amount offered in the sure option is the same as the odds represented in the gambling option. The sure choice was either presented as an option to “keep” or “lose” a portion of the initial endowment. All “keep” trials had equal but opposing “lose” trials.

In the financial-based risky-choice framing paradigm, participants are presented with an initial endowment of money and then asked to choose between taking a sure portion (presented as either a gain or loss) of the money or choosing to gamble to either win or lose the entire initial amount (as illustrated in **Figure 1**). Participants received a virtual financial endowment at the beginning of each frame ranging from $25 to $100 in increments of $25 to allow for assessment of framing bias in both small and large dollar amounts. They were then asked to choose between a sure option (framed as either a gain or loss) versus a gamble option. For trials framed as a gain, sure options were presented as an option to “keep” a portion of the initial allotment of money (e.g., “Keep $20” of $50). Loss trials were presented as a sure option to “lose” a portion of the initial allotment of money (e.g., “Lose $30” of $50). The alternative option was to gamble – to either keep or lose the entire initial endowment.

The probability of keeping or losing the entire amount in the gamble was represented on screen as a pie chart. These probabilities in the pie chart ranged from 20 to 80% in increments of 20%. Green portions of the pie chart represent the chance of winning and red represent the chance of losing. Options for each trial were presented simultaneously on the computer screen with the sure option on the left and the gamble option on the right. In each decision to be made, the percentage chance of the gamble was identical to the percentage of the initial endowment kept/lost in the sure option (e.g., a guaranteed $20 of $50 was paired with a 40% chance of keeping $50). All gain trials were equivalent to the loss trials in everything but the frame in which they were presented; for every gain trial (e.g., surely keeping $20 of $50 vs. a 40% chance of keeping the endowed $50) there was an equal but opposite loss trial (e.g., surely losing $30 of $50 vs. a 60% chance of losing the endowed $50). Susceptibility to framing was derived from a score calculated by subtracting the number of choices made with-frame (sure choices in the gain frames and gamble choices in the loss frames) from the number of choices that went against-frame (sure choices in the loss frames and gamble choices in the gain frames) for each paired trial and adding these values across all matched frames.

In accordance with the design developed by De Martino et al. (2006), “catch” trials were included in the task to assure that participants were engaged and understood the task. “Catch” trials were trials presented in an unbalanced design where gamble options were presented as a 95% chance of winning or losing the entire initial endowment alongside a sure option of keeping/losing 50% of the initial endowment. This ensured that participants had a clear indication of the best (most optimal) option to choose, namely the sure option when the gamble pie chart showed a 95% chance of losing the whole initial amount and the gamble option when the gamble pie chart showed a 95% chance of winning back the whole initial amount. “Participants who chose correctly on more than 80% of “catch” trials were assumed to be attending to and understanding the task. The data of participants were excluded if they chose incorrectly on more than 20% of “catch” trials. Excluded participants comprised less than 8% (15 out of 211 participants) of all participants tested.

### Cognitive Testing

Cognitive performance was measured on a wide array of tests, which were selected specifically to assess abilities of executive function, memory and complex attention. Cognitive measures such as abstraction, fluency, inhibition, reasoning, strategic control, immediate and delayed memory, working memory, processing speed, switching, and attention were postulated to influence framing effects in adults. **Table 2** provides a definition of each test used in the battery as well as the cognitive abilities each test measures.

### Analyses

Four conditions were created to understand participant responses in relation to framing manipulations. The conditions encompassed: (1) when participants chose the sure option when sure choices were framed as gains (gain sure), (2) the gamble option when sure choices were framed as gains (gain gamble), (3) the sure choice when sure choices were framed as losses (loss sure), and (4) the gamble option when sure choices were framed as losses. Risk-averse choices were defined as participants choosing the sure option in either the gain or loss frame. Risk-seeking choices were defined as participants choosing to gamble in either the gain or loss frame. Drawing upon prospect theory (Kahneman and Tversky, 1979), we categorized participant responses as either with-frame (defined as choosing sure options in the gain frame and gamble options in the loss frame) or against-frame (defined as choosing gamble options in the gain frame and sure options in the loss frame). It should be noted that with-frame and against-frame choices can be either risk-averse or risk-seeking. Framing effects were calculated as with-frame choices minus against-frame choices and reverse framing effects were calculated as against-frame minus with-frame choices.

Behavioral data were analyzed using the R statistic software. The mean RTs for the framing data were calculated for each frame by choice condition (i.e., Gain Sure, Gain Gamble, Loss Sure, and Loss Gamble). The main effect of frame was calculated by subtracting against-frame choices from with-frame choices. The calculated framing effect was then used as the dependent variable in a regression to understand the influence of age and cognition on framing effects. Additionally, risk-averse choices as well as risk-seeking choices were also used as dependent variables, each in their own model, to understand the influence of age and cognition on separate components of framing effects. We used general linear models (GLMs) using frame (gain and loss) and endowment ($25, $50, $75, and $100) to determine the effects of frame and endowment on participants’ choices (sure and gamble) in one GLM and reaction times in another GLM. Additionally, separate GLMs were conducted using each endowment and gambling probability category as dependent variables to understand the impact age, cognition and demographic variables had on each category.

A linear regression analyses was used to determine the effects of frame by choice conditions on framing effects. Additionally, a GLM was used to see if age, demographic variables and cognitive variables influenced framing effects, risk-averse decision, and risk-seeking decisions. Factors were first identified through step-wise variable selection and assessed by the Bayesian Information Criterion which are the factors reported on below. Interactions among cognitive and individual variables were included in the linear model. Additionally, age was also used as a covariate in regression analyses of cognitive variables on framing.

## Results

**FIGURE 2:**
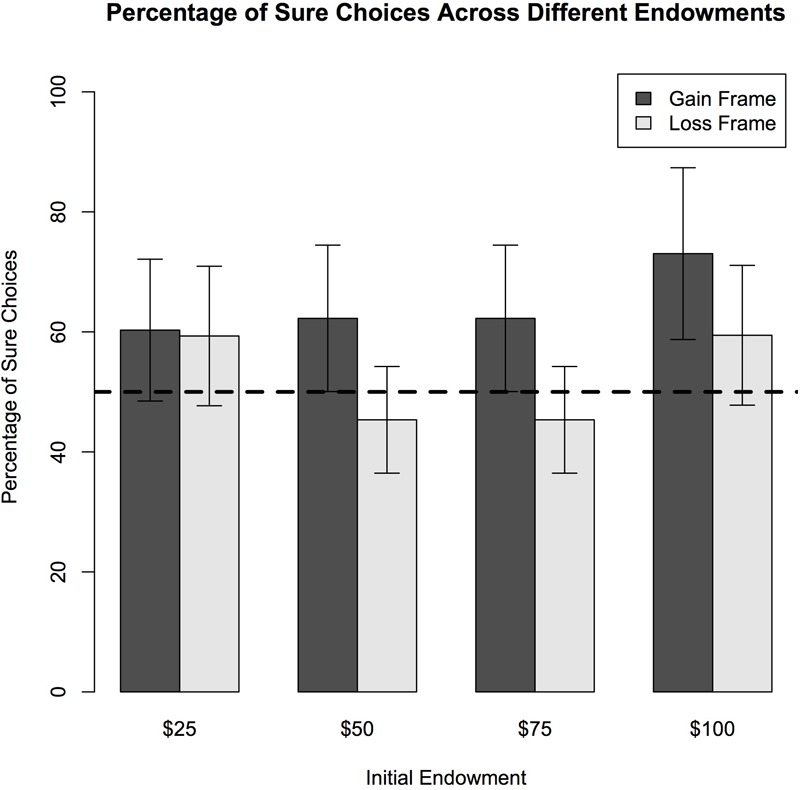
Behavioral results of decisions across varying endowments. The plot shows the percentages of trials in which subjects chose the sure option in the gain and loss frames for four different starting amounts. In a 2 × 4 (frame-by-initial amount) ANOVA analysis revealed a main effect of frame [*F*(2,193 = 17.31, *p* < 0.001]. Participants demonstrated risk-aversion (i.e., choosing the sure option) more frequently as the initial endowment increased. Increased age predicted higher sure choices only in the $100 endowment category [*F*(4,193) = 2.70, *p* = 0.008] but did not predict choices in the other endowments. Also, a main effect of endowment amounts [*F*(4,193) = 22.57, *p* < 0.001] demonstrated that participants had the highest framing effects in the $50 and $100 categories. The effect of initial endowment on sure choices was significantly greater in the gain frame than in the loss frame (the interaction of frame-by-initial amount) [*F*(3,193) = 2.28, *p* = 0.025].

**FIGURE 3:**
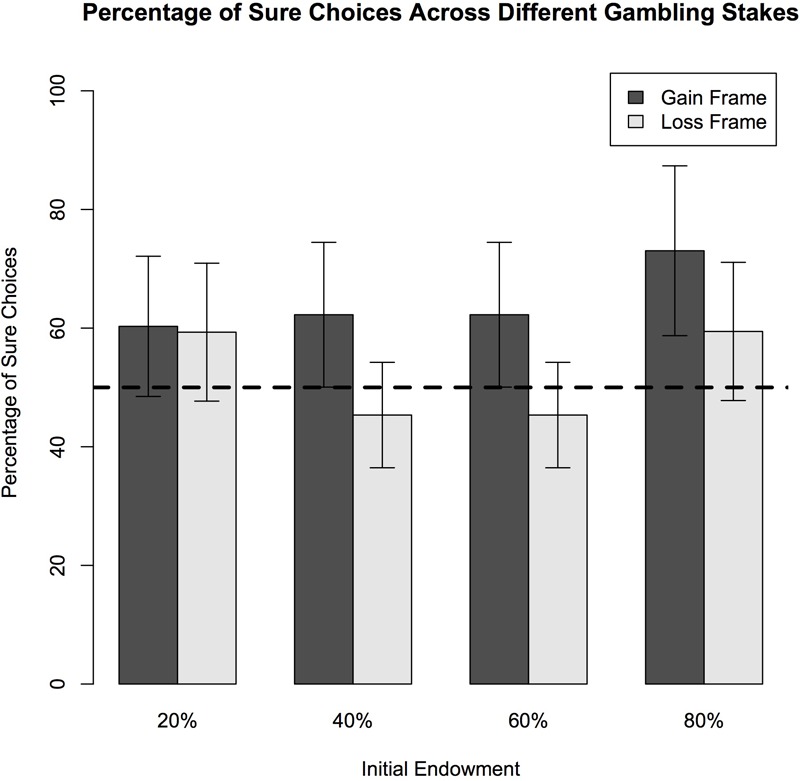
Behavioral results of decisions across varying probabilities of gambling stakes. The plot shows the percentages of trials in which subjects chose the sure option in the gain and loss frames for four different gambling probability percentages. In a 2 × 4 (frame-by-percentage) ANOVA analysis revealed a main effect of frame [*F*(2,19 = 17.31, *p* < 0.001]. Participants demonstrated risk-aversion (i.e., choosing the sure option) more frequently as the initial endowment increased. Increased age predicted higher sure choices only in the 80% probability percentage category [*F*(4,193) = 2.77, *p* = 0.026] but did not predict choices in the other probability percentages. Also, a main effect of endowment amounts [*F*(4,193) = 41.69, *p* < 0.001] demonstrated that participants had higher framing effects with increasing probability percentages. The effect of initial endowment on sure choices was significantly greater in the loss frame than in the gain frame (the interaction of frame-by-initial amount) [*F*(3,193) = 4.23, *p* = 0.007].

When evaluating the group as a whole, this sample of adults demonstrated significant framing effects during performance on this monetary-based framing task. Specifically, frame (i.e., gain vs. loss) was a significant predictor of framing effects [*F*(2,193 = 17.31, *p* < 0.001] such that participants were more likely to choose a risky option when presented with a loss and a sure option when presented with a gain (See **Table 3**). Endowment categories significantly predicted framing effects [*F*(4,193) = 22.57, *p* < 0.001] such that the lowest framing effects were present at the $25 endowment and the highest framing effects were present at the $50 endowment (See **Figure 2**) without accounting for age. Thus, when faced with choices of $25, individuals tended to select more consistent responses; whereas on endowments of $50 adults tended to make different choices (i.e., choosing the sure option in the gain frame and gamble option in the loss frame) despite the fact that many options had the same outcome. The percent chance of winning in the gamble option also significantly predicted framing effects such that the lowest framing effects were seen when the gamble option had a 20% chance of winning and the highest framing effects were seen when the gamble option had a 40% chance of winning [*F*(4,189) = 41.69, *p* < 0.001] (See **Figure 3**). Like above, this means that participants were more likely to choose the same sure or gamble option regardless of frame when the percentage chance to win the gamble was 20% and they were more likely to choose differently when the percentage chance to win the gamble was 40%. Reaction times of the framing task did not significantly predict framing effects and did not significantly differ between endowment types or percent chance of winning in the gambling. Age, however, tended to predict reaction times. Reaction times were slower with older adults, specifically in gain frame choices and risk-seeking choices, although these results were not statistically significant (See **Table 3**).

**Table 3:** Behavioral performance on framing paradigm.

Choice by frame	Average percentage of choices (SD)	Influence of age on choice	Average reaction time in seconds (SD)	Influence of age on reaction time in seconds
Gain sure	29.79% (+/-10.89%)	*t*(1,100) = 0.51, *p* = 0.60	8.43 (+/-1.40)	*t*(1,100) = –1.79, *p* = 0.08
Gain gamble	20.21% (+/-10.89%)	*t*(1,100) = -0.51, *p* = 0.60	7.20 (+/-5.95)	*t*(1,100) = 1.89, *p* = 0.06
Loss sure	24.11% (+/-11.98%)	*t*(1,100) = 0.51, *p* = 0.61	7.98 (+/-5.75)	*t*(1,100) = 0.54, *p* = 0.58
Loss gamble	25.89% (+/-11.98%)	*t*(1,100) = -0.51, *p* = 0.61	7.05 (+/-4.15)	*t*(1,100) = 1.39, *p* = 0.16
Risk-averse	53.91% (+/-22.11%)	*t*(1,100) = 0.53, *p* = 0.59	8.21(+/-8.26)	*t*(1,100) = -1.32, *p* = 0.18
Risk-seeking	46.09% (+/-22.11%)	*t*(1,100) = -0.53, *p* = 0.59	7.13 (+/-4.78)	*t*(1,100) = 1.78, *p* = 0.08
With-frame	55.68% (+/-5.97%)	*t*(1,100) = -0.08, *p* = 0.93	15.48 (+/-15.41)	*t*(1,100) = -1.24, *p* = 0.21
Against-frame	44.32% (+/-5.97%)	*t*(1,100) = 0.08, *p* = 0.93	15.18 (+/-10.67)	*t*(1,100) = 1.34, *p* = 0.18
Correct catch trials	94.18% (+/-115%)	*t*(1,100) = 0.23, *p* = 0.63	6.14 (+/-11.21)	*t*(1,100) = -0.48, *p* = 0.63

**FIGURE 4:**
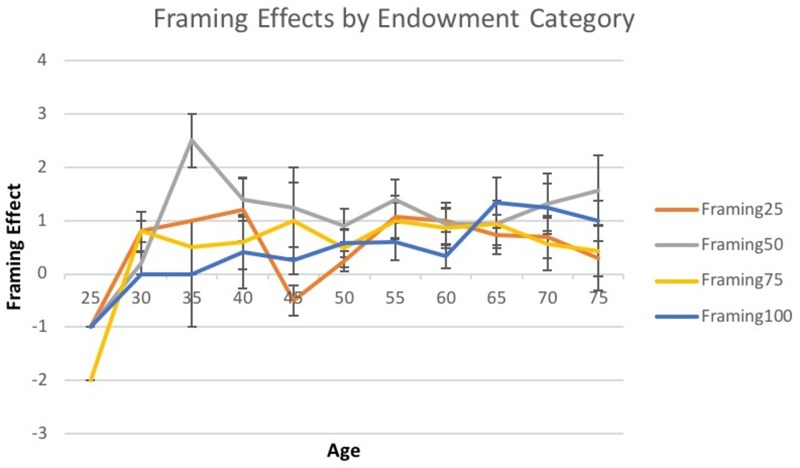
Framing effects across age by endowment category. A line graph of framing effects in each endowment category across age at 5 years intervals. The amount deviation from 0 in framing effects represents the amount of susceptibility to framing bias. Each colored line represents the mean of framing effects in a different endowment category for every 5 years. Every mean for each of 5 years also shows standard error bars. The difference of framing effects across age was only significant in the $100 category [*t*(1,100) = 2.56, *p* = 0.01].

When looking at subcomponents of the framing paradigm, such as endowment levels; some different patterns emerged on percentage chance of winning in gambles, risk-averse versus risk-seeking choices and with- versus against-frame choices. When examining framing effects across endowment categories, age significantly predicted greater framing effects in the $100 endowment category [*t*(1,100) = 2.56, *p* = 0.01] (See **Figure 4**). This pattern arose from older adults choosing more risk-seeking options in the $100 endowment, meaning that older adults were more likely to be swayed by the frame of the decision, in the highest endowment rather, than in lower endowments. In contrast, the propensity to choose with-frame or against-frame choices was not dependent on age (See **Table 3**). Framing effects analyzed as a composite score across all endowment levels did not show any significant age effects [*F*(1,193) = 2.37, *p* = 0.13].

Age did have a significant effect on the cognitive measurements of processing speed [*t*(1,193) = 8.95, *p* < 0.001], switching [*t*(1,193) = 6.52, *p* < 0.001], fluid intelligence [*t*(1,192) = -2.75, *p* = 0.007] and fluency [*t*(1,192) = -3.59, *p* < 0.001] such that increased age led to declines in these measures. Additionally, older adults had fewer years of education than younger adults [*t*(1,184) = -2.64, *p* = 0.01]; however, it is important to note that all individuals within this sample were relatively well educated with the majority (84.18%) having 16 years of education or more. There also were more females in the older group than males in this sample of participants (32.2% of the sample in adults 28–50 and 51.8% of the sample in adults 51–78) although older adults still did not differ from younger adults in framing effects when controlling for gender [*F*(2,192) = 1.47, *p* = 0.23].

**FIGURE 5:**
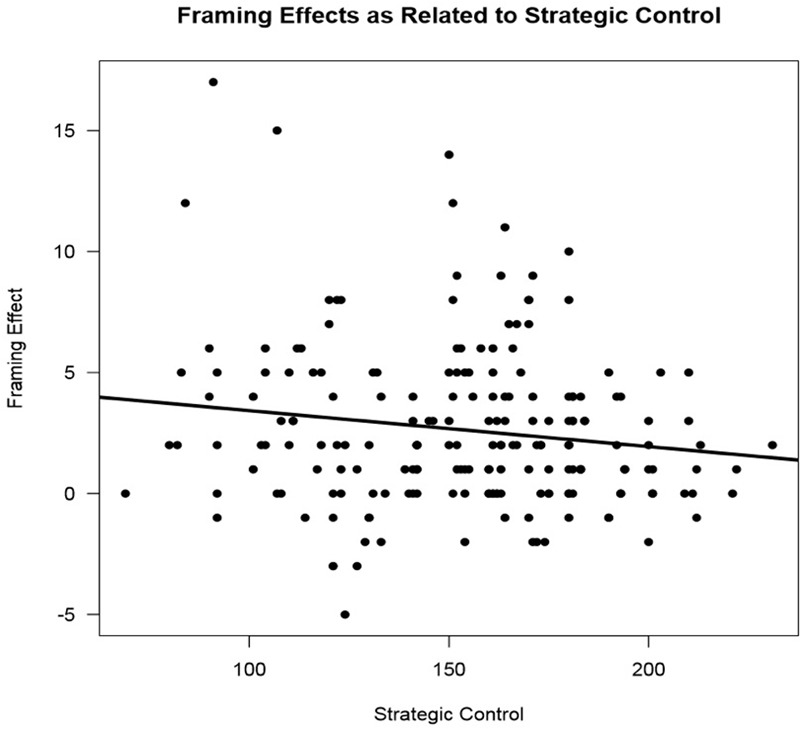
The influence of strategic control on framing effects. A scatter plot of scores on a measure of strategic control and framing effects. The amount deviation from 0 in framing effects represents the amount of susceptibility to framing bias. The solid line represents the influence of strategic control on framing effects [*F*(1,193) = 432, *p* = 0.04] which was significant.

**FIGURE 6:**
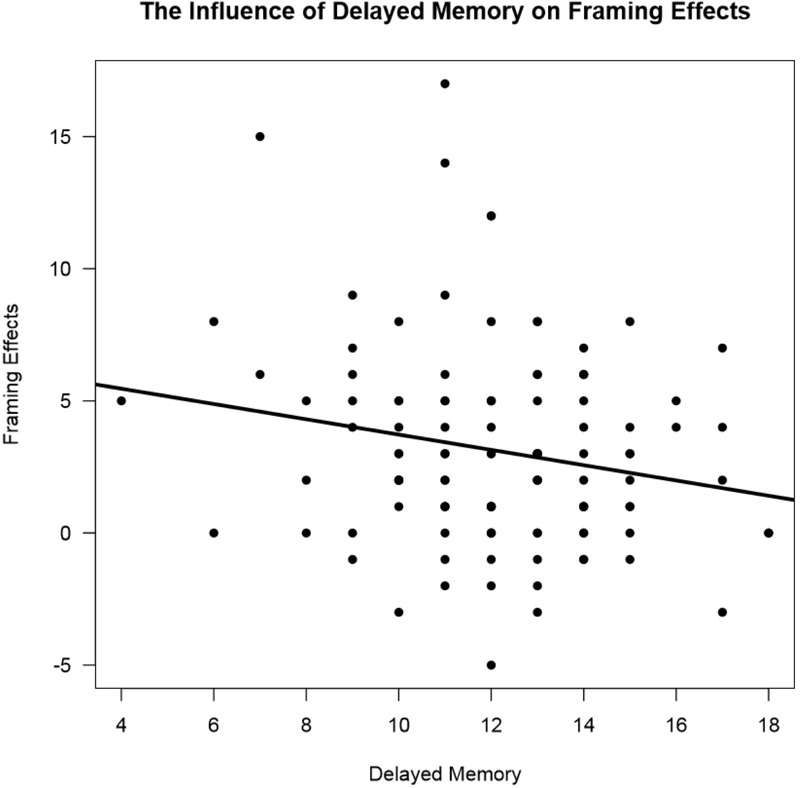
The influence of delayed memory on framing effects. A scatter plot of scores on a measure of delayed memory and framing effects. The amount deviation from 0 in framing effects represents the amount of susceptibility to framing bias. The solid line represents the influence of delayed memory on framing effects [*F*(1,193) = 4.53, *p* = 0.03] which was significant.

In measures of cognition, strategic control [*t*(1,193) = -2.08, *p* = 0.03] predicted framing scores, such that higher strategic control scores meant lower susceptibility to framing biases (See **Figure 5**). Greater delayed memory also predicted lower susceptibility to framing biases (See **Figure 6**). Additionally, the propensity for participants to make a risk-averse choice was significantly predicted by a cognitive measure of delayed memory [*t*(1,64) = 2.15, *p* = 0.03]. Also, the propensity to make a with-frame choice was significantly predicted by cognitive measures of strategic control [*t*(1,101) = -2.76, *p* = 0.006] and delayed memory [*t*(1,64) = -2.36, *p* = 0.02]. Higher strategic control and delayed memory scores predicted participants choosing against-frame choices more often than participants with low strategic control and delayed memory scores. A cognitive measure of delayed memory [*t*(1, 113) = -2.12, *p* = 0.04] also predicted framing effects, such that higher performance on a measure of delayed memory predicted fewer framing effects. Other cognitive measures did not significantly predict framing effects (See **Table 4**). In a regression analysis, age as a co-variant explained some variance attributed to strategic control [*t*(2,192) = -1.65, *p* = 0.10], however, age as a co-variant increased the significance of the delayed memory finding [*t*(2, 112) = -2.28, *p* = 0.025]. The individual demographic factors of gender and education failed to predict framing effects both on their own and when controlling for age.

**Table 4:** Cognitive factors influencing framing performance.

Cognitive domain	Cognitive test	*N*	df	Mean	Std. deviation	*t*	*p*
Executive function	Abstraction	193	1	12.38	2.5	0.84	0.40
	Fluency	146	1	46.47	11.21	-0.88	0.38
	Inhibition	87	1	11.65	2.17	-0.27	0.78
	DKEFS sorting	192	1	13.63	2.18	-0.012	0.99
	Strategic control	194	1	153.46	33.37	-2.08	0.03^∗^
Memory	Immediate memory	114	1	10.71	2.71	-0.02	0.98
	Delayed memory	114	1	12.11	2.53	-2.12	0.03^∗^
	Working memory	194	1	7.57	2.52	-1.05	0.29
Complex attention	Processing speed	194	1	25.70	8.64	1.62	0.10
	Switching	194	1	57.70	17.38	0.14	0.86
	Attention	194	1	11.66	2.39	-0.99	0.32

***^∗^**Denotes significant contribution to framing effect at p < 0.05.*

## Discussion

This study represents one of the first investigations of the influence of age across a continuous five-decade age cohort, cognition and individual factors of gender and education on financial framing effects using small amounts of money. Older adults showed greater susceptibility to framing biases when more money was at stake as compared to younger adults. In addition, older adults demonstrated lower performance on some cognitive measures of executive function and memory as compared to younger adults. As expected, higher performance on cognitive measures, specifically those of strategic control and delayed memory, predicted reduced framing effects regardless of age.

Older adults demonstrated higher framing effects when faced with $100 endowments by choosing risk-averse options when decisions were framed as gains, and choosing risk-seeking options when decisions were framed as losses. The inconsistency of choices, even though the outcomes of the decisions were equivalent, was greater in older adults as compared to younger adults in the $100 endowment category. This pattern suggests that older adults may be more vulnerable to framing biases when more money is at stake. Previous studies have similarly found that framing biases in adults became more pronounced with increased valence (i.e., amount of money at stake) due to increased loss aversion for higher amounts of money (Kahneman et al., 1991; Mobbs et al., 2006; Morewedge and Giblin, 2015). Older adults have shown increased loss aversion as compared to young adults (Johnson et al., 2006; Peters et al., 2007). The trend for increased loss aversion in older adults is theorized to be due to differences in motivation. Central motivation of young adulthood is to acquire maximum resources whereas older adults are more motivated to preserve resources (Baltes et al., 1999; Carstensen et al., 2006; Ebner et al., 2006). Another theory posits that older adults may be more susceptible to framing effects due to heightened anxiety. Anxiety is associated with greater susceptibility to the framing effect (Lauriola and Levin, 2001; Hartley and Phelps, 2012). Also, heightened anxiety has been shown to be more prevalent in older adults than younger cohorts and may contribute to stronger loss aversion in later life, particularly with greater stakes (Igartua and Cheng, 2009). Loss aversion in older adults may explain why adults prefer certain gains and losses in lower endowments but when endowments become too high the aversion to certain losses pushes older adults into making risky choices to maintain their resources. Increased framing effects in older adults for larger financial endowments may help explain why some older adults could be more susceptible to fraudulent schemes in which decisions are presented in terms of large likelihood for losses (Peters et al., 2007; James et al., 2014).

In addition to higher framing effects, older adults demonstrated lower performance on cognitive measures of processing speed, switching, abstraction, fluid intelligence, delayed memory and fluency, similar to previous evidence involving age-related declines in cognition (Park et al., 2002; Salthouse, 2010). Given that this sample of participants showed a similar pattern of age-related declines on measures of cognition as reported in prior research, it is likely that this sample represents a typical, normally aging population. Measures of cognition that did not show age-related declines were reasoning, strategic control, and inhibition. The results of this suggest that although increased age may bring on declines in some cognitive abilities, age may also show preserved functioning in other cognitive abilities (e.g., strategic control and reasoning) that support maintained decision-making performance in everyday tasks, commensurate with previous research showing preserved cognitive abilities in healthy older adults (Chapman et al., 1995, 2006; Anand et al., 2011).

In regards to cognition, we found that measures of strategic control and delayed memory significantly predicted framing effects as well as the propensity to choose with-frame or against-frame choices. This novel finding is intriguing since strategic control, by its very nature requires analytic rather than heuristic processing. One way to explain this significant link between performance on our strategic control measure and framing effects may be interpreted in light of Stanovich’s dual process model of cognition.

The dual process model by Stanovich and colleagues proposes two distinct kinds of thinking; one fast and intuitive and the other slow and deliberative (Carstensen et al., 1999; Stanovich and West, 2000; Kokis et al., 2002; Kahneman, 2011). In regards to framing effects, dual process accounts predict that individuals using fast and intuitive processing are likely to show greater framing effects than individuals who use slow and deliberative processing to approach decisions. For example, people who are automatically risk-averse when faced with loss or risk-seeking when faced with gains would be using fast thinking to process those decisions. Alternatively, people who resist the framing bias, namely are risk-averse or risk-seeking regardless of being faced with losses or gains, would be using slow and deliberate thinking to process their decisions.

Behaviorally, dual process models have had success in explaining framing effects and considerable research has described their findings in terms of dual process systems (Kim et al., 2005; De Martino et al., 2006; De Bruin et al., 2007; Kahneman and Frederick, 2007; Thomas and Millar, 2011; Murch and Krawczyk, 2013). An examination of the neural mechanisms underlying the framing effect revealed support this theory by demonstrating increased BOLD activation in the amygdala, a region associated with emotion and heuristics, and decreased vmPFC activation, a region associated with analytic thought processing, predicted greater susceptibility to framing (Rushworth et al., 2004; Deppe et al., 2005; De Martino et al., 2006). Previous studies have theorized that individual differences in aging, for example, can predict whether individuals will use automatic or deliberate processing (Deppe et al., 2005; Kim et al., 2005; Venkatraman and Huettel, 2012). Additionally, studies that have found higher executive function performance relates to reduced susceptibility to framing biases have explained their findings in terms of dual-process models (Rushworth et al., 2004). Specifically, that executive function may be an indirect measure of deliberate thinking which in turn can predict an individual’s susceptibility to framing effects.

Various measures of strategic control have shown that framing effects may be influenced by the ability to decipher and implement strategies (Kim et al., 2005; Woodhead et al., 2011; Worthy and Todd Maddox, 2011). The strategic control measure used in the present study invokes skill in discerning and employing strategies to achieve an overarching goal, which is similar to other measures of strategic ability that have been predictive of framing performance. The underlying cognitive processes associated with strategic control are effortful and align well with Stanovich’s theory of deliberate processing. The relationship between strategic control and overcoming framing effects may therefore draw upon similar deliberate processes.

Additionally, higher performance on a cognitive measure of delayed memory predicted reduced framing effects. Other components of memory such as greater working memory and immediate memory have been found to reduce framing effects as well (Castel et al., 2002; Cokely and Kelley, 2009; Lighthall et al., 2014). In relation to Stanovich’s dual-process model, memory has been theoretically divided into two systems with relative ease. The heuristic processing system has been tied to associative retrieval and pattern completion components of memory (Smith and DeCoster, 2000; Wixted and Mickes, 2010). The analytic processing system in relation to memory involves the intentional retrieval of explicit or symbolically represented rules to guide behavior (Smith and DeCoster, 2000). Delayed memory may be more of an analytic process that determines the value of what information is necessary to remember for later and apply for salient decisions. Given that delayed memory may represent an analytic form of processing information, it’s relation to overcoming framing biases would concur with Stanovich’s dual process theory on framing effects (Stanovich and West, 2008; Evans and Stanovich, 2013). Future studies should examine more extensive components of memory related to analytic processing to understand their contribution to framing effects.

## Conclusion

This study represents the first known examination of the influence of age across a continuous five-decade age cohort, cognition and individual differences on financial framing effects using small amounts of money. The key results can be summarized by the following points. First, older adults demonstrated more susceptibility to framing biases when faced with larger amounts of money. As expected, older adults also demonstrated declines in cognitive abilities as has been previously well established. Despite declines in certain cognitive abilities, greater performance on measures of strategic control and delayed memory predicted reduced susceptibility to framing biases, regardless of age. Both strategic control and delayed memory may represent cognitive measures of analytic processing which is necessary for overcoming framing biases.

Although this study provided findings that further the field of decision-making and cognition in aging, there were inherent limitations. One limitation is that the sample had a small range of education so these results may not be generalizable to people with other socioeconomic statuses. Additionally, this study used an experimental financial framing paradigm and not measures of real-life decision-making. It would be informative to understand how experimental measures of decision-making relate to real-life measures of decision-making.

Future studies are warranted to examine whether these cognitive abilities directly relate to measures of analytic processing and how older adults can be cued to use analytic processing to avoid framing effects in potentially fraudulent scams. An examination of the underlying neural mechanisms of framing effects across a large age span could better elucidate the types of cognitive processes (e.g., analytic versus heuristic) necessary for avoiding framing biases and how those processes change with age. Additionally, research should also be directed toward understanding how framing effects are impacted by early stages of disease and cognitive decline in early dementia as opposed to healthy aging, as has been suggested in an earlier study (Perez et al., 2014).

Previous research has suggested increased age predicts declines in decision making competence and biases, however, some differences shown with increased age can be attributed differences in cognitive ability, cognitive functioning, experience, and health among other factors. This research is particularly important because the findings caution against attributing impaired decision-making to age alone. Instead, identifying what capacities contribute to reduced capabilities in decision-making require continued study with precise and accurate characterizations across a compendium of factors, including but not limited to age and cognition. The present results provide valuable foundational evidence to further explore the complex neurobiological factors that are associated with financial framing effects.

## Ethics Statement

This study was carried out in accordance with the recommendations of The University of Texas at Dallas Institutional Review Board with written informed consent from all subjects. All subjects gave written informed consent in accordance with the Declaration of Helsinki. The protocol was approved by The University of Texas at Dallas Institutional Review Board.

## Author Contributions

AP: lead author, conception and design of the work, acquisition, analysis and interpretation of the work, and approval of the manuscript. JS: analysis and interpretation of the work, revisions of the work, and approval of the manuscript. LK: conception of design for the work, analysis and interpretation of the work, revisions of the work, and approval of manuscript. EV: acquisition of the work, revisions of the work, and approval of the manuscript. SC: conception and design of the work, interpretation of data, revising of work, and approval of manuscript.

## Conflict of Interest Statement

The authors declare that the research was conducted in the absence of any commercial or financial relationships that could be construed as a potential conflict of interest.
